# Occlusion-Based Three-Dimensional Craniofacial Anthropometric and Symmetric Evaluation in Preadolescences: A Comparative COHORT Study

**DOI:** 10.3390/jcm12155017

**Published:** 2023-07-30

**Authors:** Gloria Chen, Emma Yuh-Jia Hsieh, Shih-Heng Chen, Betty C. J. Pai, Ching-Yen Tsai, Sheng-Wei Wang, Pang-Yun Chou

**Affiliations:** 1 Department of Plastic and Reconstructive Surgery, Craniofacial Research Center, Chang Gung Memorial Hospital, Chang Gung University, Taoyuan 333, Taiwan; vian907gc@gmail.com (G.C.);; 2 Division of Craniofacial Orthodontics, Department of Dentistry, Craniofacial Research Center, Chang Gung Memorial Hospital, Taoyuan 333, Taiwan; emmajia@cgmh.org.tw (E.Y.-J.H.);; 3 Department of Biomedical Engineering, National Yang-Ming University, Taipei 11217, Taiwan; 4 Department of Mechanical Engineering, Chang Gung University, Taoyuan 333, Taiwan

**Keywords:** class II malocclusion, class III malocclusion, anthropometry, symmetry, preadolescence, three-dimensional images

## Abstract

Background: The importance of early diagnosis of pediatric malocclusion and early intervention has been emphasized. Without use of radiation, 3D imaging holds the potential to be an alternative for evaluating facial features in school-aged populations. Methods: Students aged 9 and 10 years were recruited. We performed annual 3D stereophotogrammetry of the participants’ heads. A total of 37 recognizable anatomical landmarks were identified for linear, angular, and asymmetric analyses using the MATLAB program. Results: This study included 139 healthy Taiwanese children with a mean age of 9.13, of whom 74 had class I occlusion, 50 had class II malocclusion, and 15 had class III malocclusion. The class III group had lower soft-tissue convexity (*p* = 0.01) than the class II group. The boys with class II malocclusion had greater dimensions in the anteroposterior position of the mid-face (*p* = 0.024) at age 10. Overall asymmetry showed no significance (*p* > 0.05). Heat maps of the 3D models exhibited asymmetry in the mid-face of the class II group and in the lower face of the class III group. Conclusion: Various types of malocclusion exhibited distinct facial traits in preadolescents. Those with class II malocclusion had a protruded maxilla and convex facial profile, whereas those with class III malocclusion had a less convex facial profile. Asymmetry was noted in facial areas with relatively prominent soft-tissue features among different malocclusion types.

## 1. Introduction

Early intervention for malocclusion in children has drawn the attention of medical specialists due to its impact on craniofacial morphology [1]. The importance of early diagnosis of preadolescence malocclusion has been emphasized [1]. Generally, craniofacial assessment could be performed simply through radiology, with lateral cephalogram being the standardized modality used for orthodontic diagnosis, treatment planning, and decision making in adults. However, avoiding redundant radiation in healthy children has been advocated widely [2]. Three-dimensional (3D) scanning of the soft tissue has been an effective method for pre-operative planning and simulation for orthognathic surgery, due to the correlation of soft-tissue characteristics with the craniofacial bone structure [3]. Additionally, it also prevents the unnecessary exposure of children to radiation. Thus, 3D analysis holds the potential to be an alternative for evaluating facial features in school-aged populations.

With the full eruption of permanent maxillary and mandibular incisors, children aged 9 to 10 maintain a stable vertical relationship of the dental features [4]. Nevertheless, without going through the prepubertal and pubertal growth spurt [5], specific facial features are not as visually noticeable as they are in adults. Recognizing certain growth trends in different occlusal groups at an early age allows early orthodontic management and may prevent worsening of lifelong temporomandibular joint health, airway development, facial morphological traits, and tooth alignment caused by malocclusion [1,6,7,8].

Although studies have reported correlations between various malocclusion types, craniofacial morphology, temporomandibular disorders, and soft tissue asymmetry [9,10,11,12,13,14], limited studies have conducted soft-tissue analysis. Moreover, no study has yet examined the correlation between 3D soft-tissue features, facial asymmetry, and different malocclusion types, specifically in a healthy pediatric population. Growing individuals all exhibit a typical growth pattern throughout their preadolescence [15], yet occlusion is regarded as a dynamic rather than a stable interrelationship between facial structures [16]. Therefore, the diversity in craniofacial development increases the unpredictability of growth in school-aged children with differing types of malocclusion [17].

This study aims at the possibility of early diagnosis of three types of malocclusion in Taiwanese preadolescents, using 3D soft-tissue imaging.

## 2. Methods

### 2.1. Study Design

The study (2201900438B0) was approved by the Ethics Committee for Human Research, Taoyuan Chang Gung Memorial Hospital, Taiwan. This observational study evaluated the facial morphology of school-aged children with various types of malocclusion by performing linear and angular measurements and examining facial symmetry. Children were randomly recruited from elementary schools in Taiwan on a voluntary basis after written informed consent was obtained from their parents. A multidisciplinary craniofacial team examined the orthodontic parameters and body height and weight of participants. The 3dMD head system (3dMD LLC, Atlanta, GA, USA) was used for imaging.

### 2.2. Subjects

Healthy Taiwanese elementary school students aged between 9 and 10 years were enrolled in this study. An experienced orthodontist evaluated the clinical parameters of the participants to ensure accurate grouping. Children with known craniofacial conditions, including congenital craniofacial anomalies and a history of craniofacial trauma or surgery, were not included in the study. A 3D scan was obtained annually with the participants being instructed to maintain a neutral facial expression during the scan. Children who did not meet the eligibility criteria and those for whom low-resolution 3D images were obtained were excluded from the study.

### 2.3. D Scanning Technique

The participants’ detailed data were obtained by performing 3D stereophotogrammetry. The 3dMD head system was used to capture 3D images of the participants. This optics-based system contains multiple modular machine vision cameras that enable comprehensive 360° head and face images to be obtained. The 3dMD Vultus software (v. 2.2, 3dMD Inc., Atlanta, GA, USA) was used to generate real-time soft-tissue images for analysis [18].

### 2.4. Landmarks

**Figure 1:**
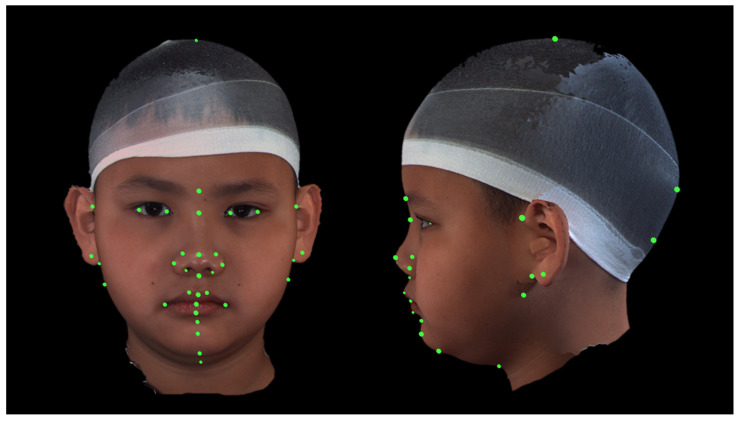
Placement of landmarks in two views. A total of 37 landmarks of recognizable anatomic structures were placed manually for the measurement-based analysis.

Two raters manually identified 37 landmarks of recognizable facial soft-tissue structures in each 3D image by using the 3dMD software (3dMD Vultus, 3dMD, Atlanta, GA, USA). A total of 14 parameters, 8 linear and 6 angular, were included in the image analysis (Figure 1 and Table 1).

The vertical dimension of the mid-face was evaluated by determining N-Sto (mid-face vertical measurement), N-Sn (total nasal height), and Sn-Sto (upper-lip vertical measurement). The anteroposterior position of the maxilla was determined by calculating the distance from Sn to N-Pg. The nasolabial angle was measured as ∠Cm-Sn-Ls.

**Figure 2:**
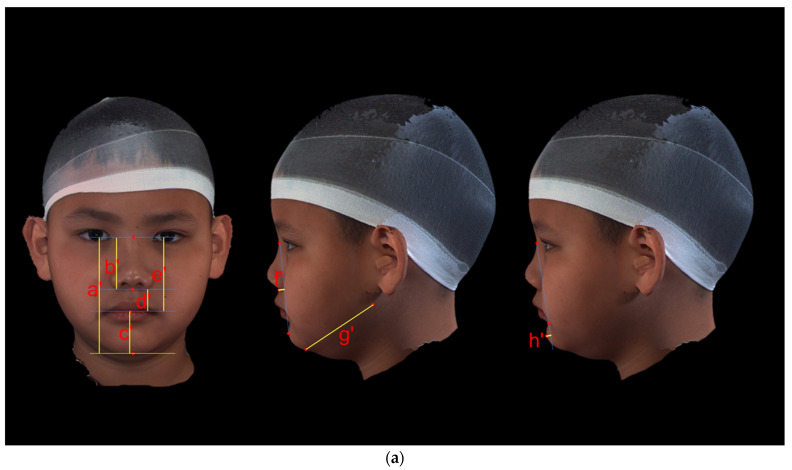

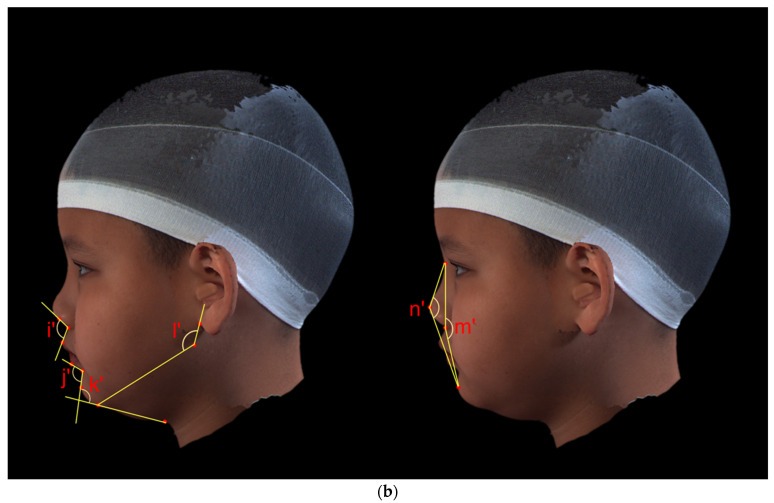
(**a**) Parameters. (**Left**) Vertical measurements of the face: (a’) N-Gn, (b’) N-Sn, (c’) Sto-Gn, (d’) Sn-Sto, and (e’) N-Sto. (**Middle**) Anteroposterior position of the subnasal region: (f’) Sn to N-Pg; length of the mandibular body: (g’) Go-Gn. (**Right**) Anteroposterior position of the pogonion: (h’) Pg to N-B. (**b**) Parameters. (**Left**) (i’) Nasolabial angle: ∠Cm-Sn-Ls; (j’) labiomental angle: ∠Li-B-Pg; (k’) lower lip to the submental plane angle: ∠B-Pg: Cer-Gn; (l’) gonial angle: ∠OBi-Go-Gn. (**Right**) (’m) Facial convexity: ∠N-Sn-Pg; (n’) full soft-tissue convexity: ∠N-PRn-Pg.

The vertical dimension of the lower face was evaluated by determining Sto-Gn (lower face vertical measurement). Go-Gn is the vertical measurement of the mandibular body. Pg to N-B represents the anteroposterior position of the pogonion. The labiomental angle was measured as ∠Li-B-Pg. The lower lip to submental plane angle was calculated as ∠B-Pg: Cer-Gn. The submental plane and soft-tissue gonial angle were measured as ∠OBi-Go-Gn. N-Gn (total facial vertical measurement), ∠N-Sn-Pg, and ∠N-PRn-Pg were used to evaluate the overall facial dimension (Figure 2a,b and Table 2).

### 2.5. Asymmetry

Asymmetry of the entire craniofacial structure and face was analyzed by calculating the difference between the distance of each point on the left side from the origin and the distance of the point’s corresponding point on the right from the origin.

**Figure 3:**
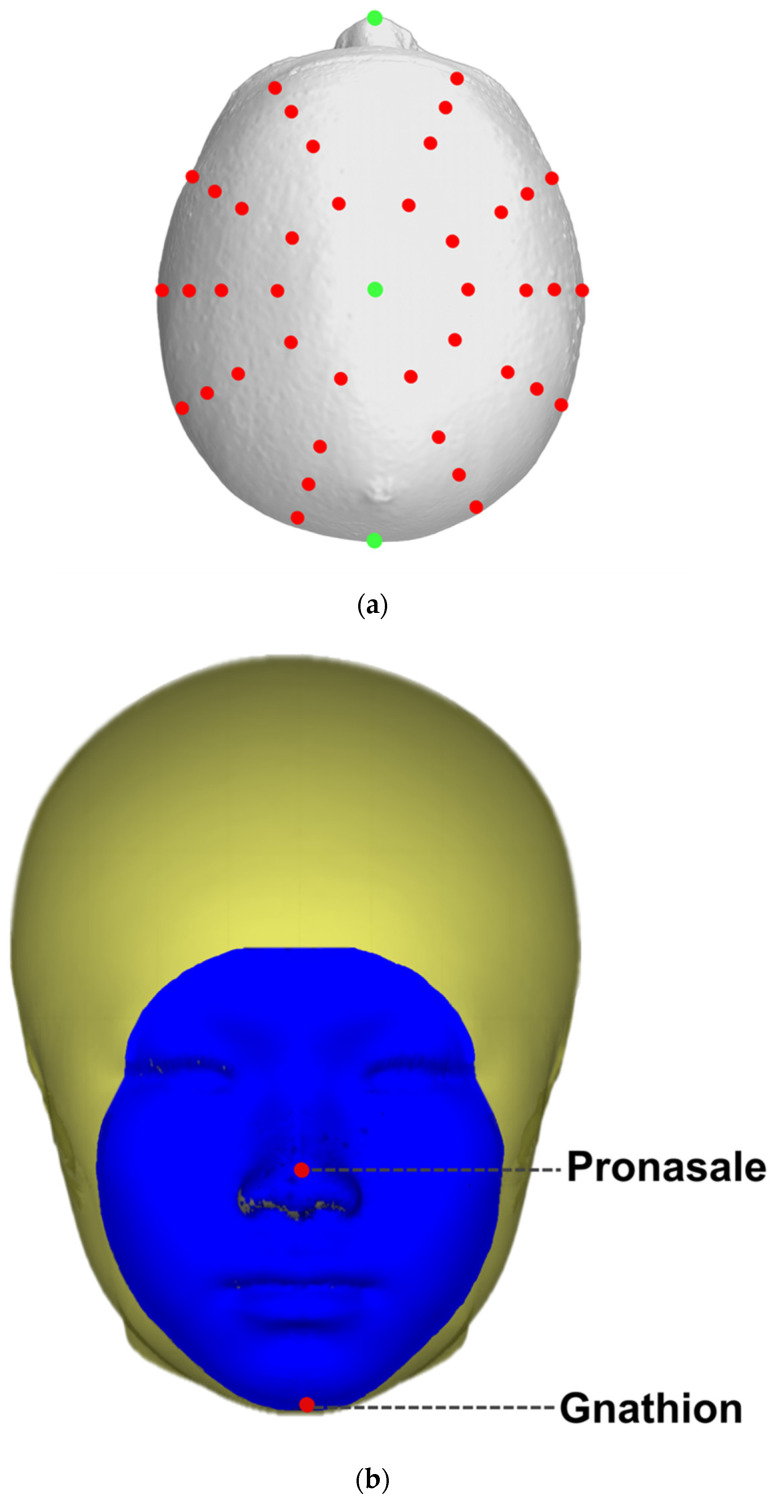
(**a**) Additional 40 digitally constructed landmarks (red) for the asymmetry analysis. (**b**) Defining the facial area (blue). Distances to the pronasale less than or equal to the distance to the gnathion are drawn as the facial area.

To examine craniofacial asymmetry, additional landmarks, evenly distributed into layers, were placed around the skull, with 10 landmarks in each layer encircling the Y axis (Figure 3a). Areas in which the distance to the pronasale position was less than or equal to the distance to the gnathion position were included in the facial area (Figure 3b).

A perfectly symmetrical craniofacial model was constructed using a customized template and MATLAB (v. 9.11, MathWorks, Natick, MA, USA).

**Figure 4:**
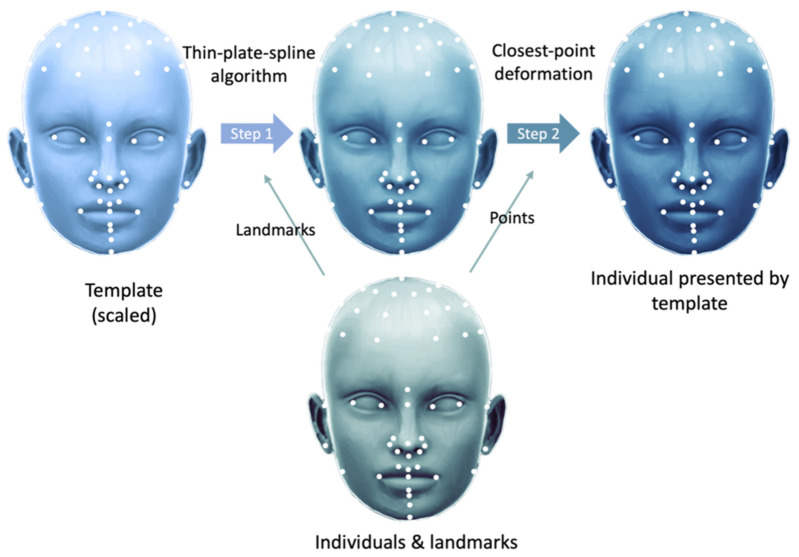
Application of the template to participants. The thin-plate splines algorithm and closest-point deformation were used on a perfectly symmetrical template for application to individual participants.

We adjusted the size of the template for each participant as required. A thin-plate spline algorithm was used on the basis of paired landmarks. Subsequently, closest-point deformation was performed to determine the point on the template correspondent with the nearest point on the 3D model (in Euclidean distance; Figure 4).

### 2.6. Statistical Analysis

All measurements were statistically analyzed using the MATLAB program. The correlations between the class I and class II occlusal group, between the class I and class III occlusal group, and between class II and class III malocclusion were determined using the unpaired two-sample *t* test. Differences in facial features and asymmetry among the three malocclusion types were examined using analysis of variance (ANOVA). A *p* value of <0.05 was considered statistically significant.

## 3. Results

A total of 139 healthy Taiwanese children were recruited to the study. Among them, 74 were in the class I occlusal group (38 boys and 36 girls), 50 had class II malocclusion (24 boys and 26 girls), and 15 had class III malocclusion (4 boys and 11 girls). The mean age of the participants was 9.13 years (Table 3).

We randomly selected 31 3dMD images from each occlusal group to determine the interobserver agreement between two raters. The method error was examined by calculating the intraclass correlation coefficient, which ranged from 0.75 to 0.97, indicating moderate to high reliability and reproducibility (Supplementary Table S1).

### 3.1. Landmark Analysis

One-way ANOVA revealed significant differences among the three malocclusion groups in the Sn to N-Pg distance in the boys (*p* = 0.024) and the N-Sn distance in the girls (*p* = 0.048) within the middle portion of the face (Table 4). We noted significant intergroup differences in the N-Sn-Pg angle (*p* = 0.029) and N-PRn-Pg angle (*p* = 0.006) in the boys. The N-PRn-Pg angle significantly differed among the three groups in general (*p* = 0.02). No significant linear relationship was noted for any parameter in the lower portion of the face (Table 5).

The Li-B-Pg angle significantly differed between the class I and class II occlusal groups (*p* = 0.046; Table 5). The N-Sn-Pg angle (*p* = 0.037) and N-PRn-Pg angle (*p* = 0.01) significantly differed between class II and class III malocclusion (Table 6). None of the parameters significantly differed between the class I and class III occlusal groups.

Although a significant association was not noted between the N-Sto distance and occlusion, a significantly smaller vertical N-Sn distance was observed. The average N-Sn distances in the girls were 43.53, 42.26, and 44.93 mm in the class I, class II, and class III malocclusion groups, respectively. However, the differences in N-Sto distance among the three groups were not significant; the N-Sto distance represents the overall vertical dimension of the mid-face. The Sn-Sto distance significantly differed (*p* = 0.007) between the boys and girls in the class I occlusal group (mean = 20.54 and 19.15 mm, respectively). The mean Sn to N-Pg distances in the boys were 6.49, 6.76, and 4.62 mm in the class I, class II, and class III malocclusion groups, respectively. We noted significantly lower and higher mean Sn to N-Pg distances in the class III and class II groups, respectively, in comparison with the class I group (Table 4). The mean N-Sn-Pg angles in the boys were 163.55°, 163.04°, and 167.98° in the class I, class II, and class III occlusal groups, respectively, whereas the mean N-PRn-Pg angles in the boys were 137.57°, 135.91°, and 140.65°. The angle was increased in the class III group but decreased in the class II group. Regardless of sex, the average N-PRn-Pg angles were 137.26°, 133.41°, and 138.85° in the class I, II, and III malocclusion groups, respectively (Table 6). A more acute Li-B-Pg angle was noted in the class II malocclusion group than in the class I occlusal group, with the mean values being 137.54° and 142.25°, respectively (Table 5).

### 3.2. Asymmetry Analysis

The mean height, anteroposterior length, width, and volume of the head were 226.85 mm, 181.50 mm, 160.82 mm, and 3486.68 mm^3^ in the class I group; 228.18 mm, 182.66 mm, 164.83 mm, and 3591.72 mm^3^ in the class II group; and 228.82 mm, 181.75 mm, 165.22 mm, and 3649.91 mm^3^ in the class III group, respectively (Table 7).

The mean asymmetry values of the skull and face were 2.49 and 0.95 mm in the class I group; 2.15 and 0.88 mm in the class II group; and 1.50 and 0.76 mm in the class III group, respectively. No significant interaction (*p* > 0.05) was discovered between malocclusion type and overall facial asymmetry in the study (Table 7).

**Figure 5:**
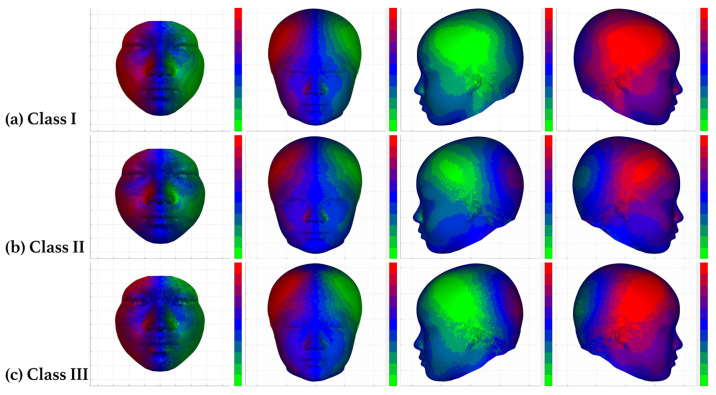
Average asymmetry heat map of the class I (**a**), II (**b**), and III (**c**) occlusion groups. From left to right, for each group the 3D heat map is presented in the facial frontal view, the craniofacial frontal view, the left lateral profile view, and the right lateral profile view. The asymmetry level of the model is represented with colors and their corresponding values. The more vivid the color the more asymmetric exhibits in the area. In the class II malocclusion group, the lower face has greater symmetricity, with patent asymmetry focused in the middle face. In the class III malocclusion group, asymmetry can be seen mainly in the lower face, with a wider area of symmetry in the middle face, specifically around infraorbital region.

Significant difference in asymmetry was not noted among the three malocclusion types. However, the symmetry noted in the 3D model revealed several morphological traits in the malocclusion groups (Figure 5). The heat map demonstrated asymmetricity in four different views in each occlusal groups’ 3D model: the facial frontal view, the craniofacial frontal view, the left lateral profile view, and the right lateral profile view. Symmetric areas mostly remained focused along the midline of the face. However, the lower face of the class II group had a greater area of symmetricity, while in the class III group, symmetricity in the middle face extended slightly into the infraorbital region (Figure 5b,c). In the class II group, an overall wide area of mild asymmetry was noted in the middle face extending to the bilateral exocanthions and the lateral portion of the oral region, which was distributed around the orbital, infraorbital, zygomatic, and parotid–masseteric regions. However, moderate asymmetricity was noted in the upper half of the buccal region (Figure 5b).

In the class III group, the orbital and infraorbital regions of the middle face and lateral part of the mental region in the lower face exhibited mild asymmetry. Moderate to severe asymmetry was noted in the zygomatic, parotid–masseteric, and temporal regions in the 3D model (Figure 5c).

## 4. Discussion

In this observational study, we examined the facial features of individuals aged 9–10 years with various types of malocclusion by performing noninvasive 3D stereophotogrammetry and compared these features among the three malocclusion types. The study included children who had fully erupted central and lateral incisors in both the maxilla and mandible. Due to well-constructed occlusal contact relation between the maxillary and mandibular permanent incisors, the participants held stability in facial features [4]. The results revealed a correlation between soft-tissue facial features and malocclusion type. Facial features are regarded as the combination of several skeletal and dental components [19]. We observed specific facial characteristics through landmark and asymmetry assessments among the three malocclusion groups.

The majority of the children in this study were in the class I occlusal group (53.2%), with the next largest group that for class II malocclusion (36.0%), and the smallest group being the class III malocclusion group (10.8%). Likewise, the pooled global prevalence of the mixed dentition stage was highest for the class I occlusal group (54% ± 12%), followed by class II malocclusion (29% ± 11%) and class III malocclusion (6% ± 2.9%); the distribution was reported to vary widely among different races and regions [20].

There was a stronger association between malocclusion and the mid-face linear measurements than those of the lower face in this study. Accordingly, we observed a more prominent maxilla in the class II group and a less protrusive maxilla in the class III group. However, prior lateral cephalometric and 3D scanning analyses demonstrated markedly increased and decreased mandibular length in patients with class III and class II malocclusion, respectively [9,10,11,12,21]. This difference may be attributed to variation in the developmental rate among the three malocclusion types [11,12]. Prepubertal individuals exhibited greater growth annually in the middle third of the face, whereas pubertal individuals demonstrated chin protrusion over time [5].

In terms of angular parameters, facial and soft-tissue convexity were smaller in the class III group and larger in the class II group than in the class I group. Facial and soft-tissue convexity are common analytical methods to examine the facial profile [22,23]. These parameters enable the analysis of facial characteristics without interference from the effect of soft-tissue thickness related to body weight [24]. Neither soft-tissue nor facial convexity differed significantly in the female participants. A reduction in facial convexity indicates a relatively retrusive maxilla or a protruded mandible, with less prominence in the mid-facial area, which may lead to facial concavity [21]. However, as a possible result due to the slower growth in the lower third of the face in preadolescences [5], facial concavity was not observed in participants in the present study. Other studies have observed a retrognathic maxilla in patients with class III malocclusion and maxillary prognathism in patients with class II malocclusion [21]. Craniofacial features in various malocclusion types are associated with sexual dimorphism mainly during pubertal and postpubertal periods [25]. Decreased facial convexity, increased maxillary retrusion, and mandibular protrusion were previously noted in male patients with class III malocclusion [11,12].

The labiomental angle is formed by the transitional region from the lower lip to the soft-tissue pogonion and is a crucial element of an individual’s aesthetic profile [26]. An acute angle of the labiomental region was reported to be strongly correlated with lower face height [27]; thus, a relatively small vertical length of the lower face can be inferred in the class II malocclusion group in this study. Common soft-tissue traits of the class II malocclusion population may be recognized as a deepened and exaggerated labiomental fold and procumbent lower lip [27].

Symmetry refers to correspondence in size, morphology, and relative position on the bilateral sides of the face [28]. The perfectly symmetrical face is rarely observed in nature and is mostly a theoretical concept [28]. A certain degree of asymmetry is normal and acceptable in an aesthetically pleasing face [29]. However, considerable facial asymmetry is a problem that individuals with dentofacial deformities have in common [30]. Although our findings revealed no significant differences in the overall asymmetricity of the face and skull among the three malocclusion groups, asymmetry of the whole face can be neutralized by various symmetric features of each proportion of the face. Patients with class II malocclusion present with a protruding maxilla and retrusion in mandible [9,10], causing a prominent facial feature around the middle facial area, thus enhancing the asymmetry around the middle portion of the face and the symmetry in the mental region. Symmetry and asymmetry were evenly dispersed in both the middle and lower facial areas in our participants with class III malocclusion. However, since the chin is considered a major facial feature for determining a balanced facial profile [31], asymmetry in the lower facial region may appear more visually severe and can be exaggerated when a facial expression is made.

Distinct facial traits in one of three malocclusion types can be observed early in preadolescence by the application of 3D image analysis. With 3D soft-tissue scanning being radiation-free and child-friendly, utilizing such a technique can help detect a dental discrepancy before full skeletal maturity. By 9 years old, those with class II malocclusion had a more protruded maxilla and a convex facial profile, whereas those with class III malocclusion displayed a less convex facial profile. Facial asymmetry was observed in areas with more prominent soft-tissue features among the three malocclusion groups.

## 5. Study Limitations

This study has some limitations that should be addressed. The sample size of the subjects included in the study was small, specifically in the class III malocclusion group. Long-term follow-up investigation of the craniofacial morphology of these groups is indicated for a more comprehensive study. Furthermore, the soft-tissue traits of the face can be affected by various confounders, ranging from genetics to environmental factors. Additionally, international multi-center enrollment could be considered for a comparative outcome of growth difference in upper and lower jaws.

## 6. Conclusions

Through 3D image study, specific facial traits and growth trends can be recognized in pediatric patients with malocclusion by the age of 9. Children with class II malocclusion present a prominent midface, acute labiomental angle, and symmetricity in the lower third of the face, while the antero-posterior position in class III malocclusion is retruded and less asymmetry presents in the mid-face area.

## Figures and Tables

**Table 1:** The 37 recognizable anatomical landmarks identified on 3Dmd images.

Landmarks	Labels	Descriptions
Glabella	G	Most anterior midpoint of the fronto-orbital soft-tissue contour
Nasion	N	Most posterior midpoint of the frontonasal soft-tissue contour
Pronasale	PRn	Most anterior midpoint of the nasal tip
Subnasale	Sn	Midpoint of the nasolabial soft-tissue contour between the columella crest and upper lip
Alare right	Ala-r	Most lateral point of the alar on the right
Alare left	Ala-l	Most lateral point of the alar on the left
Columella	C	Midpoint of the columella crest
Nostril base point right	Nb-r	Lowest point of the right nostril
Nostril base point left	Nb-l	Lowest point of the left nostril
Alar superior point right	As-r	Facial insertion of the right alar
Alar superior point left	As-l	Facial insertion of the left alar
Exocanthion right	Ex-r	Outer commissure of the right eye
Exocanthion left	Ex-l	Outer commissure of the left eye
Endocanthion right	En-r	Inner commissure of the right eye
Endocanthion left	En-l	Inner commissure of the left eye
Labiale superius	Ls	Midpoint of the vermilion line of the upper lip
Labiale inferius	Li	Midpoint of the vermilion line of the lower lip
Crista philtra right	Cph-r	The right point at the crossing of the vermilion line and elevated margin of the philtrum
Crista philtra left	Cph-l	The left point at the crossing of the vermilion line and elevated margin of the philtrum
Stomion	Sto	Midpoint of the horizontal labial fissure
Cheilion right	Ch-r	Labial commissure on the right
Cheilion left	Ch-l	Labial commissure on the left
Gonion right	Go-r	The most lateral point of the soft-tissue contour of the right mandibular angle
Gonion left	Go-l	The most lateral point of the soft-tissue contour of the left mandibular angle
Sublabiale	B	The most posterior midpoint of the labiomental soft tissue contour
Pogonion	Pg	The most anterior midpoint of the chin
Gnathion	Gn	The most inferior midpoint of the chin
Otobasion superius right	OBs-r	Point of attachment of the right helix in the temporal region
Otobasion superius left	OBs-l	Point of attachment of the left helix in the temporal region
Otobasian inferius right	OBi-r	Point of attachment of the right earlobe to the cheek
Otobasian inferius left	OBi-l	Point of attachment of the left earlobe to the cheek
Lobule right	L-r	Center of the right earlobe
Lobule left	L-l	Center of the left earlobe
Vertex	V	Highest point of the head
Opisthocranion	Op	Most posterior point of the head
Occipital	O	Most anterior point of the occipital region of the head
Cervical	Cer	Midpoint of the subhyoid depression

**Table 2:** A total of 14 parameters, 8 linear and 6 angular, were included in the image analysis.

Measurements	Label	Type	Definition
Midface			
N-Sto	e’	Linear	Mid-facial vertical measurement
N-Sn	b’	Linear	Total nasal vertical measurement
Sn-Sto	d’	Linear	Upper lip vertical measurement
Sn to N-Pg	f’	Linear	Anteroposterior position of the subnasale
∠Cm-Sn-Ls	I’	Angular	Nasolabial angle
Lower face			
Sto-Gn	c’	Linear	Lower facial vertical measurement
Go-Gn	g’	Linear	Length of the mandibular body
Pg to N-B	h’	Linear	Sagittal position of the pogonion
∠Li-B-Pg	j’	Angular	Labiomental angle
∠B-Pg: submental plane	k’	Angular	Lower lip to submental plane angle
∠OBi-Go-Gn	l’	Angular	Gonial angle
Overall			
N-Gn	a’	Linear	Total facial vertical measurement
∠N-Sn-Pg	m’	Angular	Facial convexity
∠N-PRn-Pg	n’	Angular	Full soft-tissue convexity

**Table 3:** Characteristics of participants included in the study at the time of undergoing the first 3dMD analysis (*n* = 139).

Demographic Characteristics	Value
Mean age	9.13
Sex Male	
	66 (47.5%)
Female	73 (52.5%)
Occlusion classification Class I	
	74 (53.2%)
Male	38
Female	36
Class II	50 (36.0%)
Male	24
Female	26
Class III	15 (10.8%)
Male	4
Female	11

**Table 4:** Mean linear and angular measurements and their standard deviations for three malocclusion classes of the mid-face in healthy Taiwanese elementary school students at age 9 (*n* = 139). *** Statistically significant (*p* Value ≤ 0.05).

	Class I (n = 74)	Class II (n = 50)	Class III (n = 15)	*p* Value (I and II and III)	*p* Value (I and II)	*p* Value (I and III)	*p* Value (II and III)
Mid-face							
Sn to N-Pg (mm)							
Male	6.49 ± 1.84 (n = 38)	6.76 ± 1.76 (n = 24)	4.62 ± 1.50 (n = 4)	*** 0.024			
Female	5.85 ± 1.96 (n = 36)	6.12 ± 2.02 (n = 26)	6.02 ± 3.40 (n = 11)	0.87			
*p* value (male and female)	0.14	0.26	0.35				
Total	6.17 ± 1.92	6.40 ± 1.92	5.26 ± 2.54	0.19	0.51	0.14	0.08
N-Sto (mm)							
Male	63.53 ± 2.93	64.71 ± 3.14	61.68 ± 4.41	0.08			
Female	62.45 ± 3.76	61.96 ± 3.49	64.27 ± 7.05	0.44			
*p* value (male and female)	0.16	0.059	0.44				
Total	62.98 ± 3.40	63.14 ± 3.58	62.88 ± 5.68	0.96	0.81	0.93	0.84
N-Sn (mm)							
Male	43.22 ± 2.32	43.99 ± 2.16	42.15 ± 2.32	0.16			
Female	43.53 ± 2.43	42.26 ± 2.31	44.93 ± 5.49	*** 0.048			
*p* value (male and female)	0.58	0.063	0.25				
Total	43.38 ± 2.37	43.00 ± 2.39	43.43 ± 4.16	0.70	0.39	0.95	0.63
Sn-Sto (mm)							
Male	20.54 ± 1.90	21.00 ± 1.97	19.70 ± 2.26	0.31			
Female	19.15 ± 2.42	20.02 ± 2.33	19.66 ± 2.46	0.34			
*p* value (male and female)	*** 0.006	0.09	0.98				
Total	19.84 ± 2.27	20.44 ± 2.22	19.60 ± 2.25	0.29	0.14	0.82	0.28
Cm-Sn-Ls angle (°)							
Male	106.85 ± 10.50	109.58 ± 9.47	108.11 ± 13.77	0.64			
Female	104.34 ± 12.48	104.86 ± 10.73	111.43 ± 10.59	0.39			
*p* value (male and female)	0.34	0.12	0.64				
Total	105.58 ± 11.54	106.88 ± 10.38	109.64 ± 12.02	0.45	0.52	0.25	0.41

**Table 5:** Mean linear and angular measurements and their standard deviations for three malocclusion classes of the lower face in healthy Taiwanese elementary school students at age 9 (*n* = 139). *** Statistically significant (*p* Value ≤ 0.05).

	Class I (n = 74)	Class II (n = 50)	Class III (n = 15)	*p* Value (I and II and III)	*p* Value (I and II)	*p* Value (I and III)	*p* Value (II and III)
Lower face							
Sto-Gn (mm)							
Male	43.72 ± 3.72 (n = 38)	46.28 ± 4.87 (n = 24)	45.63 ± 6.35 (n = 4)	0.09			
Female	43.53 ± 4.21 (n = 36)	43.56 ± 4.72 (n = 26)	43.01 ± 4.02 (n = 11)	0.96			
*p* value (male and female)	0.84	0.05	0.40				
Total	43.63 ± 3.95	44.73 ± 4.92	44.42 ± 6.35	0.39	0.17	0.53	0.84
Go-Gn (mm)							
Male	61.45 ± 4.33	60.84 ± 4.43	60.50 ± 6.03	0.82			
Female	60.00 ± 3.64	60.26 ± 3.34	62.93 ± 2.87	0.16			
*p* value (male and female)	0.12	0.38	0.39				
Total	60.72 ± 4.04	60.51 ± 3.81	61.62 ± 4.82	0.67	0.77	0.47	0.38
Pg to N-B (mm)							
Male	2.60 ± 2.22	2.16 ± 3.29	2.19 ± 1.24	0.79			
Female	1.82 ± 1.54	1.95 ± 1.41	1.82 ± 1.64	0.93			
*p* value (male and female)	0.076	0.85	0.65				
Total	2.20 ± 1.94	2.04 ± 2.38	2.02 ± 1.39	0.89	0.68	0.74	0.97
Li-B-Pg angle (°)							
Male	140.48 ± 12.09	135.18 ± 14.60	143.79 ± 16.38	0.22			
Female	143.97 ± 11.80	139.31 ± 13.34	141.69 ± 9.53	0.32			
*p* value (male and female)	0.20	0.84	0.79				
Total	142.25 ± 11.99	137.54 ± 13.90	142.82 ± 13.16	0.11	*** 0.046	0.87	0.22
B-Pg:Submental plane (°)							
Male	121.81 ± 8.31	124.55 ± 9.19	120.89 ± 6.51	0.42			
Female	119.60 ± 9.51	119.50 ± 10.10	116.56 ± 13.27	0.78			
*p* value (male and female)	0.28	0.10	0.46				
Total	120.69 ± 8.95	121.67 ± 9.95	118.89 ± 9.98	0.62	0.57	0.51	0.38
Go angle (°)							
Male	146.85 ± 3.89	145.38 ± 4.75	144.51 ± 3.80	0.24			
Female	146.65 ± 3.80	146.38 ± 4.75	147.92 ± 2.09	0.60			
*p* value (male and female)	0.82	0.48	0.08				
Total	146.75 ± 3.82	145.90 ± 4.07	146.36 ± 3.49	0.47	0.24	0.56	0.88

**Table 6:** Mean linear and angular measurements and their standard deviations for three malocclusion classes of the overall face in healthy Taiwanese elementary school students at age 9 (*n* = 139). *** Statistically significant (*p* Value ≤ 0.05).

	Class I (n = 74)	Class II (n = 50)	Class III (n = 15)	*p* Value (I and II and III)	*p* Value (I and II)	*p* Value (I and III)	*p* Value (II and III)
Overall measurements							
N-Gn (mm)							
Male	103.80 ± 4.31 (n = 38)	106.98 ± 4.75 (n = 24)	104.16 ± 7.32 (n = 4)	0.05			
Female	102.65 ± 5.58 (n = 36)	102.02 ± 5.56 (n = 26)	104.59 ± 9.06 (n = 11)	0.62			
*p* value (male and female)	0.32	0.051	0.93				
Total	103.22 ± 4.99	104.14 ± 5.74	104.36 ± 7.81	0.59	0.34	0.49	0.91
N-Sn-Pg angle (°)							
Male	163.55 ± 4.45	163.04 ± 4.08	167.98 ± 3.68	*** 0.029			
Female	164.84 ± 4.92	163.91 ± 5.04	165.29 ± 7.19	0.71			
*p* value (male and female)	0.23	0.52	0.40				
Total	164.21 ± 4.71	163.54 ± 4.63	166.74 ± 5.50	0.10	0.43	0.08	*** 0.037
N-PRn-Pg (°)							
Male	137.57 ± 3.53	135.91 ± 2.82	140.65 ± 3.51	*** 0.006			
Female	136.96 ± 3.25	136.21 ± 3.31	136.74 ± 4.20	0.67			
*p* value (male and female)	0.43	0.74	0.09				
Total	137.26 ± 3.38	136.08 ± 3.08	138.85 ± 4.20	*** 0.02	0.05	0.13	*** 0.01

**Table 7:** Mean craniofacial norms, head, and facial asymmetry with their standard deviations in various malocclusion classes of healthy Taiwanese elementary school students (*n* = 139, mean age = 9.13 years).

	Class I (n = 74)	Class II (n = 50)	Class III (n = 15)	*p* Value
H	228.6 ± 9.81	228.82 ± 10.54	227.1 ± 9.13	0.84
L	181.38 ± 6.87	182.19 ± 7.74	180.58 ± 8.25	0.71
W	160.98 ± 10.10	164.77 ± 12.70	161.00 ± 8.78	0.15
AH	2.32 ± 1.14	2.18 ± 1.13	1.85 ± 0.85	0.32
AF	0.94 ± 0.51	0.88 ± 0.35	0.82 ± 0.36	0.60
V	3544.74 ± 325.32	3611.73 ± 455.37	3519.81 ± 352.09	0.55

## Data Availability

Freely available to the community.

## Supplementary Materials

The following supporting information can be downloaded at: https://www.mdpi.com/article/10.3390/jcm12155017/s1, Table S1: Intraclass correlation coefficients for the linear and angular measurements.
