# Supplementary material for: Occlusion-Based Three-Dimensional Craniofacial Anthropometric and Symmetric Evaluation in Preadolescences: A Comparative COHORT Study
Source: J Clin Med. 2023 Jul 30;12(15):5017. doi: 10.3390/jcm12155017 (PMC10419555; doi:10.3390/jcm12155017)
Supplement: Supplementary file 1 [file jcm-12-05017-s001.zip › jcm-2511242-supplementary.pdf]

Supplementary Table S1.

Parameter	Intraclass correlation co-efficient	95% confidence interval	
		Lower limit	Upper limit
Sn to N-Pg (mm)	0.895	0.793	0.948
N-Sto (mm)	0.908	0.789	0.958
N-Sn (mm)	0.835	0.082	0.952
Sn-Sto (mm)	0.814	0.544	0.931
∠Cm-Sn-Ls (°)	0.895	0.622	0.960
Sto-Gn(mm)	0.852	0.717	0.925
Go-Gn (mm)	0.756	-0.09	0.941
Pg to N-B (mm)	0.966	0.890	0.989
∠Li-B-Pg (°)	0.935	0.649	0.978
∠B-Pg:Submental plane (°)	0.947	0.700	0.983
∠Go (°)	0.755	0.189	0.925
N-Gn (mm)	0.946	0.834	0.978
∠N-Sn-Pg (°)	0.904	0.773	0.956
∠N-PRn-Pg (°)	0.905	0.812	0.953
H	0.946	0.864	0.977
L	0.962	0.921	0.982
W	0.942	0.868	0.975
AH	0.766	0.541	0.889
AF	0.877	0.736	0.946
V	0.830	0.647	0.924
